# Supplementary material for: From genome to phenome: Predicting multiple cancer phenotypes based on somatic genomic alterations via the genomic impact transformer
Source: Pac Symp Biocomput. Author manuscript; Available in PMC 2021 Jan 1. (PMC6932864)
Supplement: 1 [file NIHMS1061137-supplement-1.pdf]

Supplementary information

S1. *Data pre-processing of SGAs and DEGs*

We obtained SGA data, including SMs and SCNAs of 4,468 tumors consisting of 16 cancer types[a] directly from TCGA portal[22] and Firehose browser of the Broad Institute[b]. For SMs: We considered all the non-synonymous mutation events of all genes and considered the mutation events at the gene level, where a mutated gene is defined as one that contains one or more non-synonymous mutations or indels. For SCNAs: TCGA network discretizes the gene SCNA into 5 different levels: homozygous deletion, single copy deletion, diploid normal copy, low copy number amplification, and high copy number amplification. We only included genes with homozygous deletion (potentially significant loss of gene function) or high copy number amplification (potentially significant gain of gene function) for further analysis, and filtered out the other three types of not-so-significant SCNAs. Therefore, we collectively designated all SGAs affecting a gene using the name of the gene being perturbed. Note that the preprocessing step of SMs and SCNAs excluded the obvious tumor passenger SGAs, since the functions of these mutated genes are not or only slightly perturbed. The remaining SGAs have the potential of being cancer drivers, such as oncogenes with gained functions, or tumor suppressor genes with lost functions. After processing genomic data from TCGA, we used a binary variable in a "one-hot" vector to indicate the genomic status of a gene. For example, we represented the genomic status of *TP53* as 1, if it is perturbed by one or more of SM/SCNA events in a tumor.

Gene expression data were pre-processed and obtained from the Firehose browser of the Broad Institute. We determined whether a gene is differentially expressed in a tumor by comparing the gene's expression in the tumor against a distribution of the expression values of the gene in the corresponding tissue-specific "normal" or control samples. For a given cancer type, assuming the expression of each gene (log 2 based) follows a Gaussian distribution in control sample, we calculated the p-values by determining the probability of observing an expression value from control distribution. Following the practice in previous work,[16] if the p-value is equal or smaller than 0.005, the gene is considered as differentially expressed in the corresponding tumor. However, if a DEG is associated with an SCNA event affecting it, we remove it from the DEG list of the tumor.

[a]Instead of single cancer types, we used all the available samples of various cancer types, to find the common signaling mechanisms SGAs in cancer. In addition, the GIT model benefits from the large scale dataset. The heterogeneity of different cancer types was stratified by the additional cancer type feature as input to the model.

[b]http://gdac.broadinstitute.org/

S2. *Gene2Vec algorithm implementation*

While gene embeddings can be directly learned using the GIT model, it has been shown in the field of NLP that the pre-trained word embeddings can significantly improve the performance in other related NLP tasks.[12,14] Such pre-trained word embeddings can capture the knowledge of co-occurrence pattern of the words in languages and exhibit sound semantic properties: words of similar semantic meanings are close in embedding space, e.g., $\mathbf{e}_{\text{“each”}} \approx \mathbf{e}_{\text{“every”}}$. We therefore propose an algorithm called "Gene2Vec" to pre-train the gene embeddings, which is closely related the skip gram word2vec[12] pre-training algorithm. The biology rationale behind Gene2Vec algorithm is that we are able to portrait the co-occurrence pattern of SGAs in each tumor, i.e., mutually exclusive mutations,[26] using gene embeddings and gene context embeddings.

Given the gene embedding $\mathbf{e}_g$ of an SGA-affected gene g and context embedding of any possible SGA-affected gene c': $\mathcal{V} = \{\mathbf{v}_{c'}\}_{c' \in \mathcal{G}}$, where $\mathcal{G}$ is the set of all possible SGA-affected genes, the skip gram paradigm assumes the probability that an alteration in gene c happens together with the alteration in gene g within a tumor with probability:

$$\Pr\left(c \in \text{Context}(g) \mid g\right) = \frac{\exp\left(\mathbf{e}_g^{\intercal}\mathbf{v}_c\right)}{\sum_{c' \in \mathcal{G}} \exp\left(\mathbf{e}_g^{\intercal}\mathbf{v}_{c'}\right)}. \tag{S1}$$

We used the negative sampling (NS) technique to approximately maximize the log-likelihood of skip gram, which would otherwise be computationally expensive to optimize if directly following Eq. (S1). Algorithm 1 shows implementation of Gene2Vec.

Data: Genomic alterations in each tumor: $\mathcal{T} = \{T_i = \{g_{i1}, g_{i2}, ..., g_{im(i)}\}\}_{i=1,2,...,N}$.

Result: Pretrained gene embedding of each gene:

$\mathcal{E} = \{\mathbf{e}_g \in \mathbb{R}^n\}_{g\in\mathcal{G}}$.

Context gene embeddings:

$\mathcal{V} = \{\mathbf{v}_g \in \mathbb{R}^n\}_{g\in\mathcal{G}}$.

$f(g) \leftarrow \frac{1}{Z}\sum_{i=1}^{N} \mathbb{1}(g \in T_i),\ g \in \mathcal{G}$; `// Gene frequency`

$f_n(g) \leftarrow \frac{1}{Z_n} f(g)^{3/4},\ g \in \mathcal{G}$; `// Normalized frequency`

$\mathbf{e}_g \sim U\left(-\frac{0.5}{n}, \frac{0.5}{n}\right)^n,\ \mathbf{v}_g \leftarrow 0^n,\ g \in \mathcal{G}$; `// Initialize gene embeddings and context embeddings`

while *not converges* **do**

  $l \leftarrow 0$; `// Total loss of a mini-batch samples`

  for $b = 1, 2, ..., batch\_size$ **do**

    $g \sim f$; `// Sample a gene`

    $g_c \sim \text{Context}(g\ ;\ \mathcal{T})$; `// Sample a context gene`

    $g_{nr} \sim f_n,\ r = 1, 2, ..., R$; `// Sample negative context genes`

    $l \leftarrow l + \text{NSLoss}\left(g, g_c, \{g_{nr}\}_{r=1}^{R}\ ;\ \mathcal{E}, \mathcal{V}\right)$; `// Update`

  end

  $(\mathcal{E}, \mathcal{V}) \leftarrow (\mathcal{E}, \mathcal{V}) - \eta \cdot \frac{\partial l}{\partial(\mathcal{E}, \mathcal{V})}$; `// Gradient descent`

end

Function `Context`$(g\ ;\ \mathcal{T})$

  $P_c \leftarrow U\left(\{g_c \mid g_c \in T_i, g \in T_i\}_{i=1,2,...,N}\right)$; `// Uniform distribution on sequence of adjacent mutations`

return P_c

Function `NSLoss`$(g, g_c, \{g_{nr}\}_{r=1}^{R}\ ;\ \mathcal{E}, \mathcal{V})$

  $l \leftarrow \log\sigma\left(\mathbf{e}_g^{\intercal}\mathbf{v}_{g_c}\right) + \sum_{r=1}^{R}\log\sigma\left(-\mathbf{e}_g^{\intercal}\mathbf{v}_{g_{nr}}\right)$; `// Negative sampling loss of one sample`

return l

Algorithm 1: Gene2Vec algorithm to pre-train the gene embeddings using skip gram with negative sampling loss. Given the context information of somatic genomic alterations (SGAs) in each cancer patient, i.e., whether two SGAs happened together in a single tumor, we pre-trained the gene embeddings (and context gene embeddings) using similar techniques to word2vec. Skip gram was used to predict the probability of co-occurred SGAs c given a known SGA g, as explained in Equation S1. Negative sampling loss was utilized to accelerate the maximization of log-likelihood in the skip gram assumption. Instead of original mutation frequency $f(g)$, the negative sampling frequency of SGA was sub-sampled by scaling to $f(g)^{3/4}$. In practice, the step size η in mini-batch gradient descent was decayed after training for every epoch to converge fast and prevent overfitting. Note that $\mathcal{E}$ here is defined slightly different from that in the main context, which contains both gene and cancer type embeddings.

S3. *Mathematical details of multi-head self-attention mechanism*

For all SGA-affected genes $\{g\}_{g=1}^{m}$ and the cancer type s of a tumor t, we first mapped them to corresponding gene embeddings $\{\mathbf{e}_g\}_{g=1}^{m}$ and a cancer type embedding $\mathbf{e}_s$ from a look-up table $\mathcal{E}=\{\mathbf{e}_g\}_{g\in\mathcal{G}}\cap\{\mathbf{e}_s\}_{s\in\mathcal{S}}$, where $\mathbf{e}_g$ and $\mathbf{e}_s$ are real-valued vectors. From the implementation perspective, we treated cancer types in the same way as SGAs, except the attention weight of it is fixed to be "1".

The overall idea of producing the tumor embedding $\mathbf{e}_t$ is to use the weighted sum of cancer type embedding $\mathbf{e}_s$ and gene embeddings $\{\mathbf{e}_g\}_{g=1}^{m}$ (Fig. 1b) :

$$\mathbf{e}_t = 1\cdot\mathbf{e}_s + \sum\nolimits_g \alpha_g\cdot\mathbf{e}_g = 1\cdot\mathbf{e}_s + \alpha_1\cdot\mathbf{e}_1 + ... + \alpha_m\cdot\mathbf{e}_m. \tag{S2}$$

The attention weights $\{\alpha_g\}_{g=1}^{m}$ are calculated by employing multi-head self-attention mechanism, using gene embeddings of SGAs $\{\mathbf{e}_g\}_{g=1}^{m}$ in the tumor (Fig. 1c):

$$\alpha_1, \alpha_2, ..., \alpha_m = \text{Function}_{\text{Attention}}(\mathbf{e}_1, \mathbf{e}_2, ..., \mathbf{e}_m). \tag{S3}$$

The attention function $\text{Function}_{\text{Attention}}$ is implemented as a sub-network. In the case of single-head attention, there is only one single head parameter $\boldsymbol{\theta}_j$, and the unnormalized weights $\{\beta_{g,j}\}_{g=1}^{m}$ can be derived as follows:

$$\beta_{g,j} = \boldsymbol{\theta}_j^{\intercal}\cdot\tanh(W_0\cdot\mathbf{e}_g),\ g = 1, 2, ..., m, \tag{S4}$$

which are further normalized to single-head weights $\{\alpha_{g,j}\}_{g=1}^{m}$:

$$\alpha_{1,j}, \alpha_{2,j}, ..., \alpha_{m,j} = \text{softmax}(\beta_{1,j}, \beta_{2,j}, ..., \beta_{m,j}), \tag{S5}$$

where softmax function is defined as : $\alpha_g = \exp(\beta_g)/\sum_{g'=1}^{m}\exp(\beta_{g'})$. In the case of multi-head attention, there exist h different parameters $\Theta = \{\boldsymbol{\theta}_j\}_{j=1}^{h}$. Then multiple attention weights of each gene embedding are generated following Eq. (S4,S5) and summed up to be the final multi-head attention weight:

$$\alpha_g = \sum\nolimits_{j=1}^{h}\alpha_{g,j} = \alpha_{g,1} + \alpha_{g,2} + ... + \alpha_{g,h},\ g = 1, 2, ..., m. \tag{S6}$$

S4. *Evaluation metrics of gene embedding space*

We designed two metrics for evaluating whether the gene embedding space is fair using the Gene Ontology (GO).[15] We mainly concentrated on evaluating whether SGA-affected genes share GO annotations in the "biological process" domain, based on the assumption that genes involved in a common biological process will likely share common functional impact. The top 1,474 frequently altered genes (affected by SGAs for more than 150 times across all the tumors in the dataset) were used for evaluation, assuming that the gene embeddings of rare SGAs may not be well learned.

NN accuracy: We first designed a metric called "nearest neighborhood (NN) accuracy" as a measure of functional similarity among genes sharing similar gene embedding. It is defined as the expectation of whether a pair of genes (g, c) that are nearest neighbors in the embedding space share at least one same GO term:

$$\text{NN accuracy} = \mathbb{E}_{\mathbf{e}_c \in \text{NN}(\mathbf{e}_g)} \left[\mathbb{1} \left(\text{GO}(g) \cap \text{GO}(c) \neq \emptyset \right) \right], \tag{S7}$$

where $\mathbb{1}$(statement) is the indicator function; GO(g) the set of GO terms assigned to gene g; NN($\mathbf{e}_g$) the set of nearest neighbors of $\mathbf{e}_g$. The expectation $\mathbb{E}$ is approximated by iterating over all possible pairs of genes. The higher NN accuracy, the functionally similar genes are more close to each other in the embedding space.

GO enrichment: Apart from the NN accuracy, which only reflects the functional similarities between two adjacent genes in embedding space, we also evaluated whether a cluster of genes close in an embedding space share GO annotations through "GO enrichment", which is defined as:

$$\text{enrichment} = \frac{\mathbb{E}_{\text{Clust}(\mathbf{e}_g) = \text{Clust}(\mathbf{e}_c)} \left[\mathbb{1}(\text{GO}(g) \cap \text{GO}(c) \neq \emptyset) \right]}{\mathbb{E}_{g,c \in \mathcal{G}} \left[\mathbb{1}(\text{GO}(g) \cap \text{GO}(c) \neq \emptyset) \right]}, \tag{S8}$$

where Clust(g) is the cluster that gene g belongs to. GO enrichment considers the functional similarities of genes that are close in the embedding space. The larger it is, the higher correlated are the GO functions and clusters (and it equals to 1 in random case).

S5. *Performance of GIT on real and shuffled data*

We plotted both F1 score and accuracy on the test set as the function of trained epochs (Figure S1 "real data"), which indicate that the model gains the capability of predicting DEGs as training proceeds, and finally reaches a stable state.

In order to validate that GIT is able to extract real statistical relationships between SGAs and DEGs, we randomly shuffled the positions of DEGs in the DEG vector of a tumor, i.e., randomly relabel DEG names, and then trained a GIT to predict DEGs from SGAs. We compared the performance of models trained with random datasets, by plotting F1 score and accuracy during the training of the models (Figure S1 "shuffled data"). Note that, since most DEGs in the data are zeros, a trivial solution is to call every DEG as 0, which can also achieve good overall accuracy and minimize loss, but that will result in a low F1 because of low recall. Indeed, the test F1 score in the DEG-permutation case drops to a very low value due to the same reason.

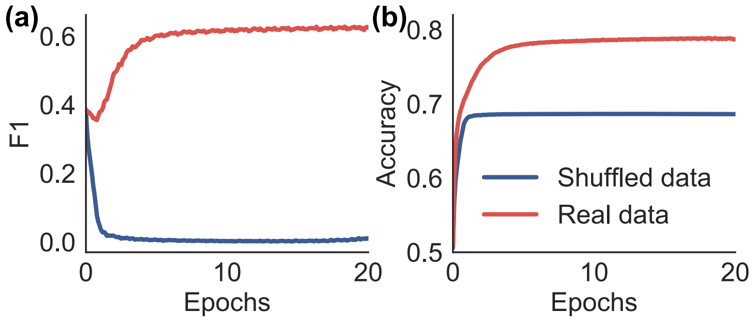

Fig. S1. **The change of F1 score and accuracy on the test set as GIT trains on real data or DEG-permuted data.**

S6. ***Enriched functions of gene clusters***

See Table S1 for the enriched functions of gene clusters. Fisher's exact test with Bonferroni correction (p-value<0.05) was implemented on genes that belong to 40 clusters. 12 clusters of genes show to be significantly involved in at least one biological process. The genes in cluster 14, referred to as "IFN pathway", was further analyzed as a case study in Sec. 3.2, which is involved in viral defense response, immune response and cell surface signaling.

Table S1. **Enriched gene ontologies in the "biological process" domain of human beings (*Homo sapiens*).**

Cluster ID	Enriched gene ontology	Enriched biological process	p-value
2	GO:0038003	Opioid receptor signaling pathway	2.09e-02
3	GO:0050911	Detection of chemical stimulus involved in sensory perception of smell	3.16e-31
	GO:0007186	G protein-coupled receptor signaling pathway	5.27e-21
4	GO:0007156	Homophilic cell adhesion via plasma membrane adhesion molecules	5.26e-03
	GO:0001568	Blood vessel development	4.64e-02
	GO:0048666	Neuron development	4.02e-02
	GO:0009653	Anatomical structure morphogenesis	3.17e-04
8	GO:0045995	Regulation of embryonic development	3.77e-02
	GO:0007155	Cell adhesion	4.28e-02
14 (IFN pathway)	GO:0033141	Positive regulation of peptidyl-serine phosphorylation of stat protein	9.07e-30
	GO:0002323	Natural killer cell activation involved in immune response	6.02e-29
	GO:0042100	B cell proliferation	1.65e-26
	GO:0043330	Response to exogenous dsrna	1.05e-25
	GO:0002286	T cell activation involved in immune response	2.22e-24
	GO:0060337	Type i interferon signaling pathway	2.93e-21
	GO:0030183	B cell differentiation	1.40e-21
	GO:0051607	Defense response to virus	2.85e-18
	GO:0007596	Blood coagulation	5.77e-14
	GO:0006959	Humoral immune response	4.82e-15
	GO:0002250	Adaptive immune response	1.61e-12
	GO:0010469	Regulation of signaling receptor activity	2.83e-12
16	GO:0050727	Regulation of inflammatory response	4.13e-02
23	GO:0003272	Endocardial cushion formation	3.61e-02
	GO:0003179	Heart valve morphogenesis	9.79e-03
	GO:0007156	Homophilic cell adhesion via plasma membrane adhesion molecules	4.87e-02
	GO:0035295	Tube development	2.32e-03
	GO:0051960	Regulation of nervous system development	4.73e-02
	GO:0007399	Nervous system development	1.88e-02
25	GO:0051179	Localization	1.19e-02
30	GO:0000904	Cell morphogenesis involved in differentiation	4.15e-02
	GO:0007155	Cell adhesion	2.93e-02
	GO:0007275	Multicellular organism development	2.21e-03
35	GO:0007156	Homophilic cell adhesion via plasma membrane adhesion molecules	1.60e-09
36	GO:0040011	Locomotion	3.42e-02
40	GO:0035589	G protein-coupled purinergic nucleotide receptor signaling pathway	4.50e-03

S7. *Top genes by attention mechanism and mutation rates*

See Table S2 for the full list of top 100 genes that are assigned by the attention mechanism. Table S3 shows the top 5 genes that are most frequently mutated in both pan-cancer and single cancer types. It serves as the control group, in comparison to the attention mechanism results (Table 3,S2; experiment group).

Table S2. **List of candidate drivers whose corresponding SGAs have top 100 highest attention weights.** Boldfaced genes are known drivers according to TumorPortal[3] and IntOGen[34] database.

Rank	Gene	Rank	Gene	Rank	Gene	Rank	Gene
1	***TP53***	26	*MUC5B*	51	*KRTAP4-11*	76	*CNTNAP3B*
2	***PIK3CA***	27	*LMTK3*	52	*CYP4F11*	77	*NKRF*
3	***RB1***	28	***AHNAK***	53	*EP400*	78	***SETD2***
4	***PBRM1***	29	***VHL***	54	***XRN1***	79	***LAMA2***
5	***PTEN***	30	***FGFR3***	55	*MBD6*	80	*AARS*
6	***CDH1***	31	*PHF20*	56	*AR*	81	*SPON1*
7	***CASP8***	32	***STK11***	57	*ANKRD30BP2*	82	*WRN*
8	***KRAS***	33	*OCA2*	58	*PRICKLE2*	83	*LHX1*
9	*SLC1A6*	34	***GATA3***	59	*RGAG1*	84	***STAG2***
10	*POMC*	35	*PCNX*	60	*KRT23*	85	*KSR1*
11	*RRN3P2*	36	*KRTAP4-9*	61	*UGT1A1*	86	*GCDH*
12	*TFAM*	37	*LRRIQ3*	62	*PARP8*	87	*E2F3*
13	*CD163*	38	*MRGPRF*	63	*TMPRSS6*	88	*PDHX*
14	*WDFY3*	39	***HSP90AA1***	64	*FMN2*	89	*CLUH*
15	*WDR44*	40	*CNTN3*	65	***CDKN2A***	90	*PRICKLE4*
16	*CYP51A1*	41	*WNK3*	66	*DIP2B*	91	*GLUD2*
17	*ADARB2*	42	*PTPRD*	67	*TBP*	92	*CROCC*
18	*C9orf53*	43	*PCDHB16*	68	*ZNF624*	93	***IDH1***
19	***BAP1***	44	*RPLP0P2*	69	*FEM1B*	94	*GRIA1*
20	*TMPRSS13*	45	*COL6A1*	70	*CDKN2B*	95	*DLG5*
21	*SV2C*	46	*TTC39B*	71	*PDE4D*	96	*SMURF2P1*
22	*MYCBP2*	47	***PGR***	72	*ISLR2*	97	*CACNA1C*
23	***MED24***	48	*TBC1D4*	73	*FLRT3*	98	*KIAA1377*
24	***CYLD***	49	*ANKRD36C*	74	*ZFAT*	99	*PTPRZ1*
25	*CYLC2*	50	*GPATCH8*	75	***SMARCA4***	100	***PCSK5***

Table S3. **Top five SGA-affected genes for Pan-Cancer and a few selected cancer types, ranked according to alteration frequency, as the control group to GIT.** The corresponding experiment group, which is the selected candidate drivers of GIT model, is shown in Table 3. The known cancer drivers according to TumorPortal[3] and IntOGen[34] are marked in bold font.

Rank	PANCAN	BRCA	HNSC	LUAD	GBM	BLCA
1	***TP53***	***TP53***	***TP53***	*TTN*	*CDKN2A*	*TTN*
2	*TTN*	***PIK3CA***	***CDKN2A***	***TP53***	*CDKN2B*	***TP53***
3	***PIK3CA***	*TTN*	*TTN*	*CSMD3*	*C9orf53*	***ARID1A***
4	*CSMD3*	*POU5F1B*	***PIK3CA***	*PCDHAC2*	***EGFR***	*DNAH5*
5	*MUC4*	*TRPS1*	*LINC00969*	*MUC16*	*MTAP*	***CDKN2A***

S8. *Survival analysis based on raw SGAs*

SGAs alone as tumor representations are not informative of predicting survival profiles. See Fig. S2 for survival analysis based on raw SGAs.

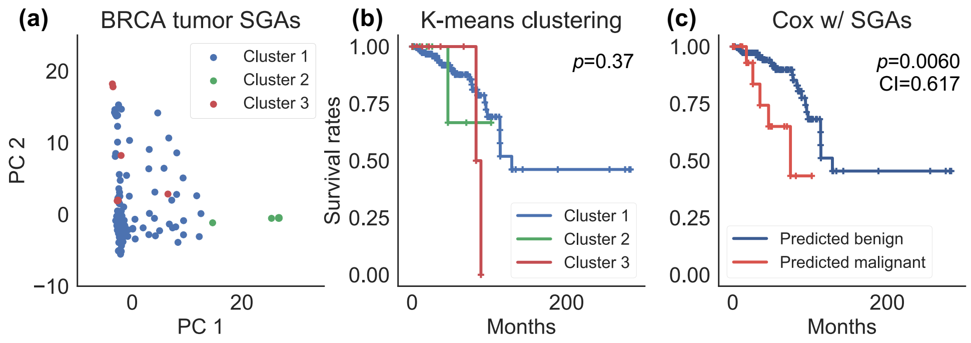

Fig. S2. **(a)** PCA plot showing k-means clustering of BRCA tumors using their SGA vectors. Most tumors merge around the origin (Cluster 1; with a small number of SGAs), while others (Cluster 2,3; with a large number of SGAs) are outliers and far away from the origin. **(b)** KM estimators and log-rank test on the three BRCA tumor groups in the SGA space. **(c)** Cox regression using SGAs (top mutated 474 genes are used).
